# Exercise Mediated Nrf2 Signaling Protects the Myocardium From Isoproterenol-Induced Pathological Remodeling

**DOI:** 10.3389/fcvm.2019.00068

**Published:** 2019-06-06

**Authors:** Gobinath Shanmugam, Anil K. Challa, Asokan Devarajan, Baskaran Athmanathan, Silvio H. Litovsky, Prasanna Krishnamurthy, Christopher J. Davidson, Namakkal Soorappan Rajasekaran

**Affiliations:** ^1^Cardiac Aging & Redox Signaling Laboratory, Division of Molecular and Cellular Pathology, Department of Pathology, University of Alabama at Birmingham, Birmingham, AL, United States; ^2^Department of Biology, University of Alabama at Birmingham, Birmingham, AL, United States; ^3^Department of Medicine, Division of Cardiology, Department of Medicine, David Geffen School of Medicine, University of California, Los Angeles, Los Angeles, CA, United States; ^4^Department of Biomedical Engineering, University of Alabama at Birmingham, Birmingham, AL, United States; ^5^Division of Cardiovascular Medicine, Department of Medicine, University of Utah, Salt Lake City, UT, United States; ^6^Center for Free Radical Biology, University of Alabama at Birmingham, Birmingham, AL, United States

**Keywords:** antioxidants, cardiac remodeling, exercise, echocardiography, isoproterenol, Nrf2

## Abstract

Although exercise derived activation of Nrf2 signaling augments myocardial antioxidant signaling, the molecular mechanisms underlying the benefits of moderate exercise training (MET) in the heart remain elusive. Here we hypothesized that exercise training stabilizes Nrf2-dependent antioxidant signaling, which then protects the myocardium from isoproterenol-induced damage. The present study assessed the effects of 6 weeks of MET on the Nrf2/antioxidant function, glutathione redox state, and injury in the myocardium of C57/BL6J mice that received isoproterenol (ISO; 50 mg/kg/day for 7 days). ISO administration significantly reduced the Nrf2 promoter activity (*p* < 0.05) and downregulated the expression of cardiac antioxidant genes (*Gclc, Nqo1, Cat, Gsr, and Gst-*μ) in the untrained (UNT) mice. Furthermore, increased oxidative stress with severe myocardial injury was evident in UNT+ISO when compared to UNT mice receiving PBS under basal condition. Of note, MET stabilized the Nrf2-promoter activity and upheld the expression of Nrf2-dependent antioxidant genes in animals receiving ISO, and attenuated the oxidative stress-induced myocardial damage. Echocardiography analysis revealed impaired diastolic ventricular function in UNT+ISO mice, but this was partially normalized in the MET animals. Interestingly, while there was a marginal reduction in ubiquitinated proteins in MET mice that received ISO, the pathological signs were attenuated along with near normal cardiac function in response to exercise training. Thus, moderate intensity exercise training conferred protection against ISO-induced myocardial injury by augmentation of Nrf2-antioxidant signaling and attenuation of isoproterenol-induced oxidative stress.

## Introduction

Nuclear factor erythroid 2 like 2 (NFE2L2/Nrf2) is the major stress response transcriptional regulator for antioxidant genes. Declined Nrf2-antioxidant signaling during aging leads to accumulation of reactive oxygen/nitrogen species (ROS/RNS) and oxidative stress, which is either causally linked or associated with numerous health problems including diabetes, cardiovascular disease, neurodegenerative conditions (Alzheimer's, Parkinson's, and Huntington), and cancer (1–4). Experimental or clinical outcomes with exogenous supplementation of antioxidants yield mixed results in various pathophysiologic settings (5–10). Also, the rationale for selection and specific action of the given antioxidants remains unclear. Hence, targeting endogenous antioxidant defense mechanisms to enhance cytoprotection may be efficacious. However, genetic approaches to augment Superoxide Dismutase (SOD, antioxidant enzyme) through transgenic overexpression (11) or targeting transcription factors (i.e., NFE2L2 or NRF2) responsible for antioxidant genes (12) have resulted in their abundance, leading to unusual redox shifts that fail to combat oxidative stress mediated anomalies (13–15). Therefore, optimal approaches that can exert transient and controlled activation of antioxidant pathways in response to toxic insults or pathological conditions are warranted. As induction of Nrf2-dependent antioxidant signaling by pharmacological agents was associated with toxic side effects (16–20), a non-pharmacologic approach may be of greater value. With these approaches, we and others have recently reported that sustained physical activity or routine exercise training upregulates Nrf2-dependent cytoprotective targets in human skeletal muscle and mouse heart (21–25). Here, we tested a hypothesis that exercise based stabilization/activation of Nrf2, and its transcriptional regulation of antioxidants protects the heart from isoproterenol-induced myocardial damage and dysfunction.

Isoproterenol (ISO), a β-adrenergic receptor agonist, has been widely accepted as an inducer of cardiac remodeling in experimental animals (26–29). Isoproterenol induces the generation of excessive ROS, leading to antioxidant depletion and oxidative stress, which cause alterations in cardiac metabolism, progressive myocardial injury and dysfunction/heart failure (28, 29). Isoproterenol-induced cardiac remodeling involves injury, necrosis, and disruption of energy reserves in cardiomyocytes, leading to cardiac dysfunction and heart failure (28, 29).

We investigated whether exercise training activates Nrf2-dependent antioxidant signaling and prevents oxidative stress when challenged with isoproterenol. More specifically, our goal is to investigate whether exercise mediated stabilization of Nrf2 can ameliorate the isoproterenol induced oxidative stress and prevent ubiquitination of proteins.

## Methods

### Reagents

RNeasy kit, reverse transcription kit, and QuantiTect SYBR Green PCR kit were purchased from Qiagen Inc., Valencia, CA. qPCR primers were designed using Primer Bank (https://pga.mgh.harvard.edu/primerbank/) or Primer BLAST website and purchased from Integrated DNA Technologies, Coralville, IA. Trans-AM Nrf2 kit (50296) for determination of Nrf2-ARE binding activity was obtained from Active Motif, Carlsbad, CA. GCLC (ab41463), NQO1 (ab34173), GSR (ab16801), and Ubiquitin (ab7780) antibodies were procured from Abcam, Cambridge, MA; rabbit anti-GAPDH (D16H11) from Cell Signaling, USA. Anti-rabbit or mouse secondary antibodies for immunoblots (horse radish peroxidase conjugated with IgG) were purchased from Vector Laboratories, Burlingame, CA. Protein Assay reagent (#500–0006) was procured from Bio-Rad, Hercules, CA. All other chemicals including oxidized glutathione, RNA*later*, meta-phosphoric acid, and isoproterenol, were purchased from Sigma-Aldrich unless otherwise stated.

### Animals

Both male and female WT (C57BL/6J) mice at the age of 6–8 months were used for the study. Mice were provided with a regular rodent diet with water *ad libitum* and maintained under a controlled temperature and humidity at 12 h light/dark cycle. The Institutional Animal Care and Use Committee (IACUC) at the University of Alabama at Birmingham approved all animal experiments, in accordance with the standards established by the US Animal Welfare Act.

### Moderate Exercise Training

**Figure 1:**
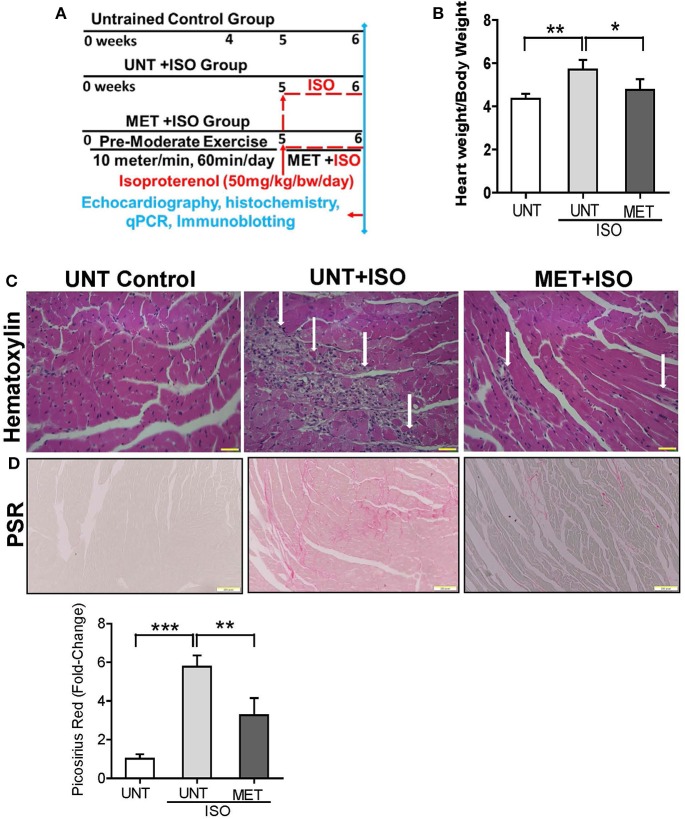
Exercise protected the heart from Isoproterenol induced cardiac injury. **(A)** Schematic diagram explaining the exercise protocol and isoproterenol administration, **(B)** Graph illustrates the heart weight/bodyweight ratio in all groups (*n* = 10/group), **(C)** Heart sections stained by hematoxylin eosin stain (*n* = 6/group), **(D)** Picrosirius red stain was used to determine the collagen deposition in isoproterenol treated hearts using Olympus light microscope at 20X magnification. *n* = 3–4/group, values are represented as mean ± SD. Significance: ^*^p < 0.05; ^**^p < 0.01; ^***^p < 0.001.

Age and sex-matched WT (C57/BL6J) mice (6–8 months old) were subjected to moderate exercise training (MET) on a treadmill for 6 weeks (60 min/day; 10 m/min; 0% grade). At the beginning of the 6th week, mice from MET trained group were selected to undergo isoproterenol administration. MET continued during ISO administration (Figure 1A).

### Isoproterenol Treatment

Dose selection is an important step in studying the isoproterenol mediated cardiac damage. Since a low dose of ISO induces hypertrophy (30–32) and a higher dose has been observed to induce severe myocardial infarction (33–37), we aimed to use a dose that induces moderate levels of oxidative stress and progressive cardiac pathology. Moreover, the concentration of isoproterenol used in this study has been previously reported by other investigators (38–40). Randomly assigned, untrained (UNT) and trained (MET) animals were subcutaneously injected (at the beginning of 6th week) with 50 mg of isoproterenol/kg.bw/day for 7 consecutive days (38–40). All the animals (UNT, UNT + ISO; MET + ISO) underwent echocardiography evaluation 24 h following the last dose of isoproterenol (22) (Figure 1A).

### Autopsy and Sample Preparation

At the end of the 6th week of MET and 1 week of isoproterenol treatment, mice were anesthetized using isoflurane and euthanized by cervical dislocation. Hearts were immediately perfused with ice cold phosphate buffered saline, removed and appropriately stored for RNA, protein, biochemical, and histological analysis. Tissues stored in RNA*later* were used for RNA isolation, and tissues were immediately flash frozen in liquid nitrogen for proteins. A small piece of the heart tissue (~20 mg) was immediately processed for GSH assay. Middle region of myocardial sections were embedded in paraffin and sectioned for histological evaluation. Slides were stained with hematoxylin and eosin to determine cardiac damage and picrosirius red (PSR) stain for collagen accumulation. Images were captured using an Olympus BX43 upright microscope.

### Non-invasive Echocardiographic Analysis of Cardiac Function

Twenty four hours after the last dose of isoproterenol, UNT, UNT+ISO, MET, and MET+ISO mice were anesthetized using 1–2% isoflurane, supplemented with oxygen and the chest area was shaved in preparation for echocardiography analyses (*n* = 5–6) using the Vevo2100 Imaging System (FujiFilm VisualSonics Inc., Ontario, Canada). A 38 MHz probe was used to capture images at maximum (50 μM) resolution. Long axis B-mode was employed for strain analysis to calculate ejection fraction and end diastole/systole left ventricular mass. The parasternal short axis M-mode was utilized in the determination of fractional shortening, wall thickness, and chamber dimension during systole and diastole. Three consecutive cardiac cycles from B and M-mode images were used for measuring for each variable (23, 41). Pulse wave Doppler imaging was performed in apical four chamber view by capturing the mitral valve blood flow to measure the diastolic function of the left ventricle. All these images were processed/analyzed using Vevolab 3.1 software to obtain the cardiac systolic and diastolic function (41).

### Trans-AM DNA Binding Activity for Nrf2

Efficiency of Nrf2-ARE binding activity was measured using a commercial Trans-AM Nrf2 kit from Active motif. Briefly, wild-type or mutated competitor oligonucleotides bearing the antioxidant response element (ARE) consensus sequence were incubated with nuclear extract (10 μg) for Nrf2 and the bound Nrf2 was incubated with an anti-Nrf2 primary antibody (100 μl of a 1:1000 dilution) for 1 h. Further, HRP-conjugated secondary antibody (100 μl of a 1:1000 dilution) was added in each well and incubated for 1 h prior to chromogenic reaction with TMB substrate and the absorbance was measured at 450 nm with a reference wave length of 655 nm using a BioTek Epoch plate reader. Incubation with normal rabbit polyclonal IgG was also performed separately to confirm the specificity of the Nrf2 antibody (12, 42).

### Isolation of RNA and Real-Time qPCR Analysis

Myocardial tissues stored in RNA-*later* from UNT, UNT+ISO, and MET+ISO mice (*n* = 3–4) were used and RNA was extracted using RNeasy mini kit (Qiagen, 74106). Then using QuantiTect reverse transcription kit (Qiagen, 205313), cDNA was synthesized using 1.25 μg RNA. Quantitative RT-PCR (qPCR) was performed using 25–50 ng cDNA with 1 pmol gene specific primer (Table 1) in a 10 μl SYBR green reaction mix (Qiagen, 204056) and amplified in a Roche Light Cycler 480 (Roche, Basel, Switzerland). Relative expression was quantified using C_t_ values, and expression fold-change was calculated by normalization to the C_t_ of housekeeping genes *Gapdh* or *Arbp1* according to the 2^−ΔΔCt^ methods (12, 23, 42, 43).

**Table 1:** List of primers used for the qRT-PCR.

**Genes name**	**Sequences (5′….3′)**
*Arbp1F*	TGAGATTCGGGATATGCTGTTGG
*Arbp1R*	CGGGTCCTAGACCAGTGTTCT
*Cat F*	GGAGGCGGGAACCCAATAG
*Cat R*	GTGTGCCATCTCGTCAGTGAA
*Gapdh F*	TGACCTCAACTACATGGTCTACA
*Gapdh R*	CTTCCCATTCTCGGCCTTG
*Gclc F*	GGACAAACCCCAACCATCC
*Gclc R*	GTTGAACTCAGACATCGTTCCT
*Gclm F*	CTTCGCCTCCGATTGAAGATG
*Gclm R*	AAAGGCAGTCAAATCTGGTGG
*Gsr F*	CACGGCTATGCAACATTCGC
*Gsr R*	GTGTGGAGCGGTAAACTTTTTC
*Gst-μ F*	CTGAAGGTGGAATACTTGGAGC
*Gst-μ R*	GCCCAGGAACTGTGAGAAGA
*Gsta F*	TGATTGCCGTGGCTCCATTTA
*Gsta R*	CAACGAGAAAAGCCTCTCCGT
*G6pd F*	TCAGACAGGCTTTAACCGCAT
*G6pd R*	CCATTCCAGATAGGGCCAAAGA
*Nqo1 F*	AGGATGGGAGGTACTCGAATC
*Nqo1R*	TGCTAGAGATGACTCGGAAGG
*Nrf2 F*	CTGAACTCCTGGACGGGACTA
*Nrf2 R*	CGGTGGGTCTCCGTAAATGG
*Sod-1 F*	AACCAGTTGTGTTGTCAGGAC
*Sod-1 R*	CCACCATGTTTCTTAGAGTGAGG
*Sod2 F*	TGGACAAACCTGAGCCCTAAG
*Sod2 R*	CCCAAAGTCACGCTTGATAGC

### Protein Isolation and Immunoblotting

Heart tissues from UNT, UNT+ISO, and MET+ISO (*n* = 3–6/group) mice at >6 months of age were homogenized using cytosolic extraction buffer [1:6 ratio, (tissue wt (mg): buffer (μl)) 10 mM HEPES, 10 mM KCl, 0.1 mM EDTA, 0.5 mM MgCl_2_, with freshly prepared 0.1 mM phenylmethylsulfonyl fluoride (PMSF), 1 mM dithiothreitol and 1% Triton X-100, pH 7.9] and centrifuged at 5,000 rpm for 5–6 min. Proteins were normalized and equal amount of cytosolic proteins from all groups were resolved on 10% SDS-PAGE and transferred to PVDF membranes and blocked in Tris Buffered Saline-Tween 20 (TBST) containing 5–10% non-fat dry milk for 2 hrs. Individual blots were then incubated overnight at 4°C or at room temperature for 2 hrs with the respective primary antibodies for GCLC, NQO1, GSR, ubiquitination, and GAPDH diluted with 2% bovine serum albumin in TBST. After two 5 min washes with TBST, the blots were incubated with horseradish peroxidase IgG (Vector Laboratories, Burlingame, CA, USA) conjugated secondary antibodies (anti-rabbit or mouse) for 1 h. Blots were then washed thrice for 10 min with TBST and treated with ECL (Pierce, Rockford, IL, USA), imaged on Amersham Imager 600 (GE Healthcare Life Sciences, Chicago, IL, USA). The immune-reactive signals were quantified by densitometry using ImageJ software and density values were normalized to GAPDH (12, 23).

### Myocardial Glutathione Levels

Myocardial levels of reduced GSH and oxidized GSH (GSSG) were assessed by a GSH detection kit from Cayman (Ann Arbor, MI, USA). In brief, heart tissues were homogenized with MES buffer and the homogenates were centrifuged at 5,000 rpm for 5 min at 4°C. An aliquot of the supernatant was used for protein estimation. An equal amount of 10% meta- phosphoric acid (MPA) was added to the remaining samples to precipitate the proteins; 100 μl of the MPA extracts were treated with triethanolamine (TEAM). After treating with TEAM, samples were mixed with 150 μl of reaction mixture cocktail (MES buffer, NADPH, glutathione reductase, DTNB) and the enzymatic-recycling assay was performed as per the manufacturer's instruction using a plate reader. GSH and GSSG standards were prepared and processed similarly to generate a standard graph (12, 23).

### Dihydroethidium Fluorescence Staining

Dihydroethidium (DHE), a lipophilic, cell permeable fluorogenic dye was used to measure the level of ROS. This dye gives off a red fluorescent signal during oxidation. Briefly, the frozen heart sections (10 μm) were incubated with 5 μg/ml of DHE in PBS in a light protected chamber maintained at 37°C incubator for 30 min. Slides were then fixed with fluoroshield mounting medium mixed with DAPI (a nuclear stain) and the images were captured by randomly selecting 3–5 fields/section which were obtained using an Olympus BX43 fluorescent microscope (12, 22).

### Statistical Analysis

All data are represented as mean ± SD. One-way ANOVA with *post-hoc* Tukey multiple comparison tests were performed. All analyses were performed using GraphPad Prism 7. Differences were considered significant at the values of ^*^p < 0.05, ^**^p < 0.01, and ^***^p < 0.001.

## Results

### Exercise Training Protects the Myocardium From ISO Induced Cardiac Injury

The UNT and MET mice received 50 mg/Kg bw subcutaneous of isoproterenol for 7 days and were assessed for myocardial remodeling, necrosis, and fibrosis using heart weight, hematoxylin-eosin, and picrosirius red staining. Administration of isoproterenol for 1 week period (7 days) in the UNT mice showed a significant increase in heart weight (HW) to body weight (BW) ratios (HW/BW) compared to the UNT control group. As expected, exercise training markedly prevented isoproterenol-mediated increase in HW/BW ratios (Figure 1B). The hematoxylin eosin (H&E) stained images from the UNT mice treated with isoproterenol displayed widespread myocardial necrosis with degeneration and obvious leukocyte infiltration (Figure 1C), whereas the MET mice treated with isoproterenol showed minimal cell death foci and diminished leukocyte infiltration. Further, we quantitatively measured the isoproterenol mediated cardiac fibrosis in all groups using picrosirius red (PSR) staining. The isoproterenol treated mice showed increased collagen in the myocardium, whereas the MET mice that received isoproterenol displayed decreased levels of collagen (Figure 1D) compared to the UNT mice. These data demonstrated that 6 weeks of exercise training protects the myocardium from isoproterenol mediated cardiac hypertrophy and damage.

### Chronic Moderate Exercise Training Ameliorates the Isoproterenol Induced Structural and Functional Changes

**Figure 2:**
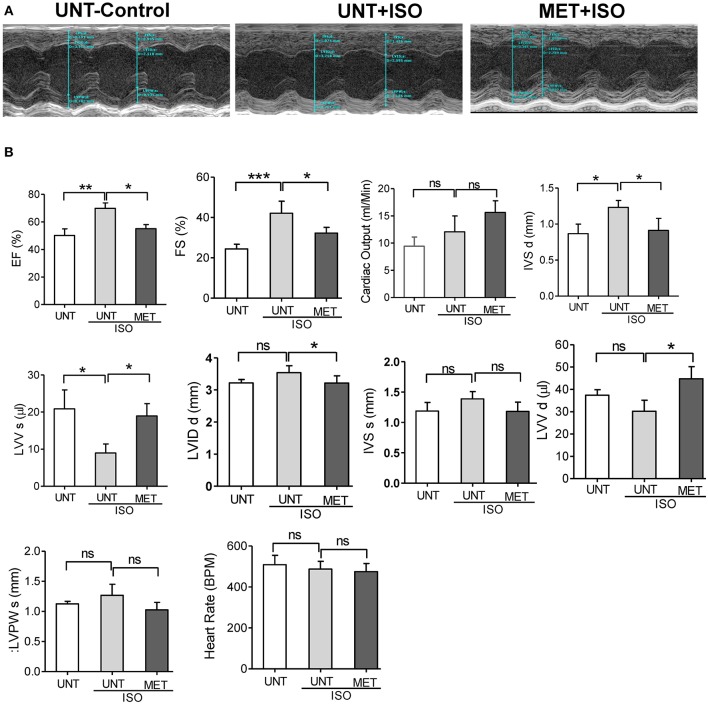
Exercise preserved the heart function from Isoproterenol induced cardiac injury. Echocardiography was performed in all the three groups on the day of sacrifice using Vevo2100 high-resolution (38 MHz) imaging system. **(A)** Representative M-mode (SAX) images from untrained control, untrained+ISO and trained+ISO mice, **(B)** cardiac functions were analyzed using LAX B-mode and SAX M-mode images and represented as a bar graph. *n* = 5–6/group, values are represented as mean ± SD. Significance: ^*^p <0.05; ^**^p <0.01; ^***^p <0.001; ns, no significance.

As exercise training protects the myocardial structure from isoproterenol induction, we assessed the cardiac function by transthoracic echocardiography. Representative parasternal short axis M-mode images displayed an abnormal cardiac structure and function in UNT+ISO mice (Figure 2A). Ejection fraction was highly increased in the UNT+ISO mice with a decrease in end-systolic ventricular volume compared to the UNT control mice. Mice subjected for exercise alone (MET) showed increased EF and LVV d (μl) (Supplemental Figures 1A,B). Exercise training significantly mitigated isoproterenol impact on end systolic volume and led to normal cardiac function (Figures 2A,B). Further, the LVIDs (left ventricular internal dimension, end-systolic), IVSd IVSd (Interventricular Septal Thickness at Diastole), and IVSs (Interventricular Septal Thickness at Systole), were significantly elevated in the UNT+ISO group compared to UNT control mice. Six weeks of chronic exercise training significantly prevented these changes induced by isoproterenol administration. These results suggest that chronic exercise training can protect the myocardium from isoproterenol induced pathological cardiac damage.

### Moderate Intensity Exercise Improves Isoproterenol Mediated Diastolic Dysfunction

**Figure 3:**
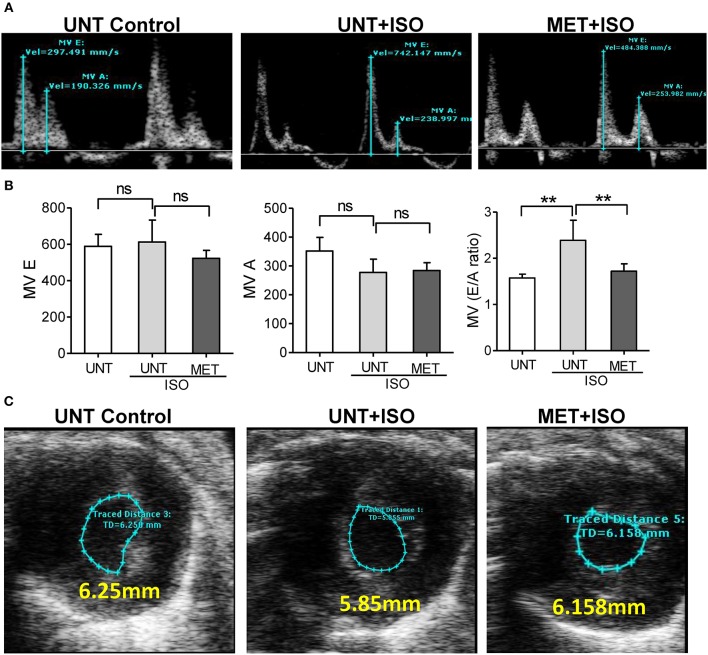
Exercise training preserved normal diastolic function in the heart treated with Isoproterenol. **(A)** The mitral valve flow pattern was recorded by pulse wave Doppler mode using Vevo2100 high-resolution echocardiography, **(B)** Mitral valve filling velocities (MV E and A) were measured using Mitral valve images and represented as bar graph, C) The representative B-Mode of SAX image displaying the level of contraction, *n* = 5–6/group, values are represented as mean ± SD. Significance: ^**^p < 0.01; ns, no significance.

Isoproterenol administration reduced the late left ventricular filling velocity (MV A) and early left ventricular filling velocity (MV E) was unchanged, leading to an increase in the atrial filling wave velocity (E/A) ratio when compared to UNT-control and MET mice treated with isoproterenol. Increased E/A ratio in UNT—isoproterenol group suggested that isoproterenol administration lead to diastolic dysfunction compared to UNT control mice. MET only subjected mice didn't show any significant changes in atrial filling velocities (Supplemental Figures 2A,B). However, the exercise trained mice receiving isoproterenol exhibited significantly reduced both MV E and MV A waves, leading to a stabilized atrial filling wave velocity (E/A) ratio equal to the UNT mice (Figures 3A,B). These results indicate that exercise training prevented the functional remodeling induced by isoproterenol administration.

### Exercise Stabilizes Nrf2 and Its Transcriptional Activity in Isoproterenol-Induced Hearts

**Figure 4:**
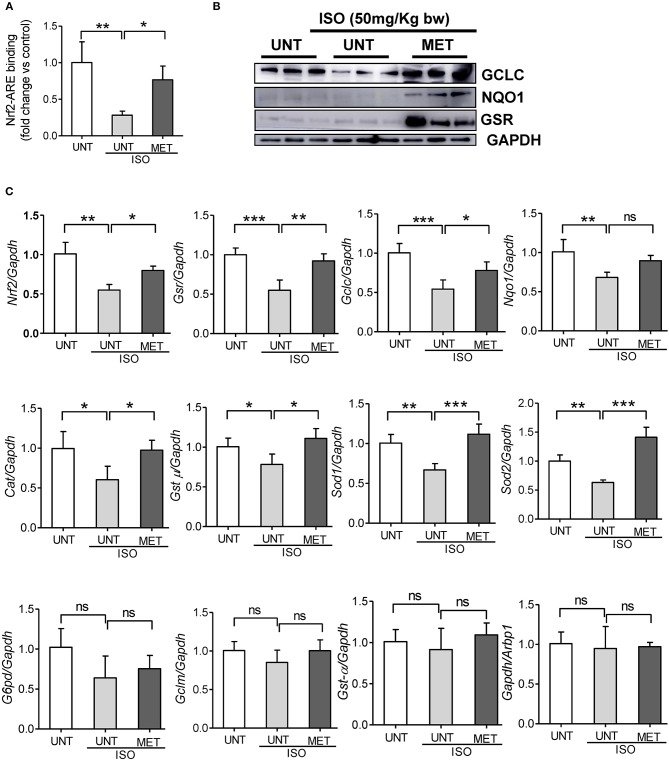
Exercise stabilized the Nrf2 binding activity and antioxidant gene expression in the heart of Isoproterenol treated mice. **(A)** Nrf2-ARE binding activity was measured in heart nuclear lysates of untrained control, untrained+ISO and trained+ISO mice using Active motif TransAM Nrf2 assay kit (*n* = 4–6/group), **(B)** Immunoblots for key antioxidants using cytosolic heart extracts of UNT, UNT-ISO and MET-ISO mice (*n* = 6/group). **(C)** Antioxidant gene levels were determined using gene specific primers by qPCR and relative gene expression was analyzed by normalizing with *Gapdh/Arbp1, n* = 4–6/group, values are represented as mean ± SD. Significance: ^*^p < 0.05; ^**^p < 0.01; ^***^p < 0.001; ns, no significance.

We assessed the Nrf2/antioxidant response element (ARE) DNA-binding ability to determine whether exercise stabilizes the Nrf2 transcriptional activity in isoproterenol induced hearts using the Trans-AM-Nrf2 binding activity. Untrained mice induced with isoproterenol showed decreased Nrf2 binding activity, whereas the exercised mice receiving isoproterenol displayed a stable Nrf2 binding activity equal to that of the untrained control mice (Figure 4A). These results confirmed that exercise induced Nrf2 was active. To confirm the Nrf2-mediated regulation of antioxidant protein expression, we measured the levels of key Nrf2-target proteins GCLC, NQO1, and GSR, using immunoblotting. Isoproterenol administration significantly reduced the expression of GCLC and NQO1 in untrained mice; however, there was no significant difference in GSR protein levels. The exercise mice receiving ISO showed significant increase in all these proteins compared to UNT and UNT-ISO groups. Additionally, to confirm Nrf2-mediated transactivation of antioxidant genes, we measured antioxidant gene expression levels by qPCR. Untrained mice receiving isoproterenol presented a significant decrease in *Nrf2, glutamate-cysteine ligase, catalytic subunit (Gclc), glutathione reductase (Gsr), NAD(P)H dehydrogenase quinone 1 (Nqo1), glutathione S-transferase, mu (Gst-*μ*,), Glucose-6-phosphate dehydrogenase (G6pd), Superoxide dismutase 1 (Sod1)* and *Sod2* (Figure 4C). Exercise trained mice were able to maintain similar levels of expression for antioxidant genes as seen in PBS-controls, while isoproterenol treatment significantly downregulated expression of most of these genes. Although the *G6pd* level was not changed in the MET+ISO group when compared to the UNT+ISO group, *Glutamate-cysteine ligase, modifier subunit (Gclm)*, and *glutathione S-transferase alpha (Gst-*α*)* were similar in all groups. These results provide evidence that exercise mediated Nrf2 stabilization tightly regulates antioxidant networks and protects the myocardium from isoproterenol mediated cardiac injury.

### Chronic Exercise Training Preserves Isoproterenol-Induced Glutathione Depletion in the Myocardium

**Figure 5:**
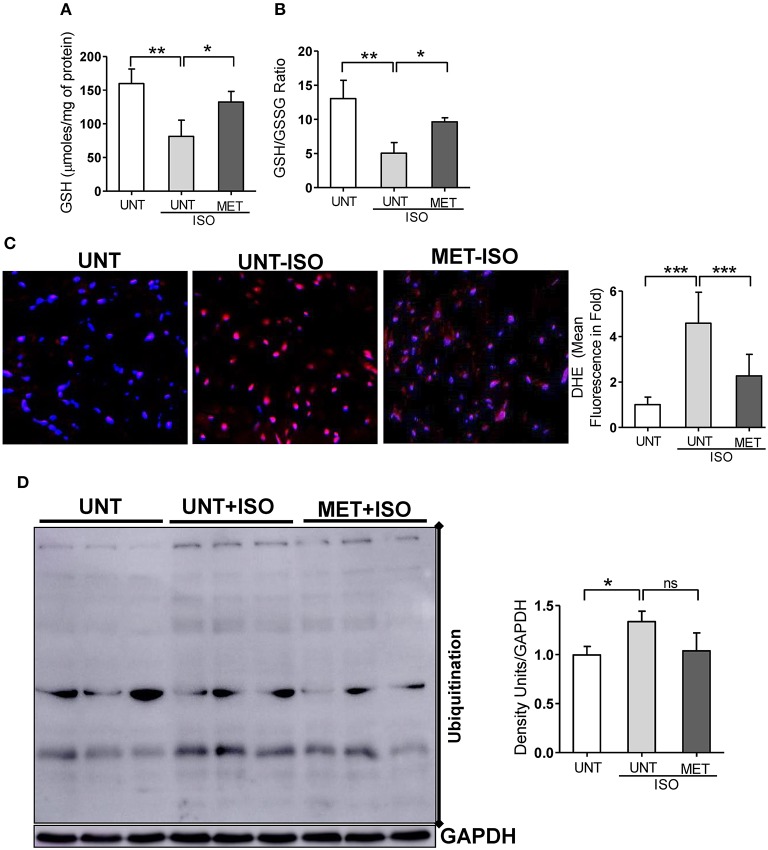
Moderate intensity exercise training improved the glutathione redox levels, reduced the ROS and ubiquitination of proteins in Isoproterenol treated hearts. **(A,B)** Tissues from all three groups (*n* = 4/group) were processed to prepare the MPA extracts and used for determining the myocardial glutathione (GSH) and oxidized glutathione levels (GSSG) by enzymatic recycling assay, **(C)** Hearts sections stained with specific fluorescence stain (DHE) for reactive oxygen species (ROS) and captured in Olympus light microscope at 40X magnification. (*n* = 4/group). **(D)** Immunoblots for ubiquitination of proteins in the Isoproterenol induced cardiomyocytes. *n* = 3–4/group, values are represented as mean ± SD. Significance: ^*^p < 0.05; ^**^p < 0.01; ^***^p < 0.001; ns, no significance.

As exercise training restored Nrf2-antioxidant signaling genes, including the genes that are involved in glutathione metabolism, we assessed glutathione, its redox ratio (GSH/GSSG), and DHE staining to measure total ROS levels in the isoproterenol induced myocardium. In response to isoproterenol induction, untrained mice showed decreased GSH and GSH/GSSG levels (Figures 5A,B), whereas the exercise training stabilized the GSH and GSH/GSSG levels in isoproterenol injected mice. Further, DHE staining showed increased ROS signals in UNT mice receiving isoproterenol; however, exercise training decreased the ROS levels induced by isoproterenol treatment (Figure 5C). These results demonstrated that exercise mediated stabilization of Nrf2-antioxidant signaling facilitates glutathione synthesis and protects the myocardium from isoproterenol mediated oxidative injury.

### Moderate Exercise Training Reduces Protein Ubiquitination in the Isoproterenol Induced Myocardium

As isoproterenol treatment showed increased ROS/oxidative stress in UNT+ISO hearts, and oxidative stress has been reported to induce post-translational modifications (PTMs) in proteins (ubiquitination) (44–46), we investigated these protein modifications using western blotting and role of exercise mediated Nrf2 stabilization in these PTMs. While increased ubiquitination of proteins were evident in UNT mice with isoproterenol (Figure 5D), when compared to UNT control mice, MET mice treated with isoproterenol showed decreased ubiquitination. These results confirmed that exercise induced Nrf2-signaling potentially reduces oxidative stress, thereby decreasing the PTMs.

## Discussion

Benefits of exercise have been widely reported to be cardio-protective (22, 23, 47–50), but the specific molecular mechanisms associated with Nrf2-dependent cytoprotection remain elusive. Here, we tested whether a non-pharmacological stabilization of Nrf2 signaling protects the myocardium from oxidative stress injury. In particular, the present investigation demonstrated that exercise training for 6 weeks stimulates Nrf2-antioxidant signaling and suppresses the isoproterenol-induced oxidative damage in the mouse myocardium. Exercise mediated Nrf2 stabilization preserves myocardial glutathione redox (GSH/GSSG) levels, reduces the ROS/oxidative stress induced by isoproterenol, and diminishes the protein modification (PTMs) in response to isoproterenol injury.

Under basal conditions, Nrf2 is redundant, but it is required in stress conditions for the transactivation of cytoprotective/antioxidant genes (51, 52). Down-regulation of Nrf2 is coupled with impaired redox-status and vascular dysfunction under hypertension (53). We previously reported that exercise mediated Nrf2 activation augments antioxidant gene expression and protects the heart from age-induced oxidative stress (22). In the present study, we have shown that exercise mediated stabilization of Nrf2 enhances antioxidant enzymes and reduces isoproterenol-induced oxidative stress and cardiac hypertrophy (Figures 4, 5). Others have reported that sulforaphane and broccoli-based Nrf2 activation protects the myocardium from Ang-II toxicity (54) and diabetes-induced cardiac dysfunction (55). MG132, a small molecule proteasome inhibitor, increases Nrf2 expression and protects the myocardium from pressure-overload-induced hypertrophy (56, 57). In the present study, we tested whether isoproterenol (ISO), a known β-AR agonist, could induce oxidative stress and cause hypertrophy and fibrosis. In particular, we investigated whether exercise mediated Nrf2 activation preserves redox levels and prevents isoproterenol-induced oxidative stress, thereby protecting the myocardium from pathological remodeling.

Isoproterenol treatment resulted in infiltration of leukocytes along with significant structural remodeling (i.e., hypertrophy) in the untrained mice. Interestingly, we observed an increased fractional shortening (FS), left ventricular cavity dimensions at diastole and systole, along with profound fibrosis in the UNT mice, suggesting an adaptive remodeling of the myocardium in response to isoproterenol induction. However, a significant decrease in LV diastolic volume in the isoproterenol-treated hearts leads to pathological remodeling (Figure 2). We also observed a significant increase in MV E/A ratio in untrained ISO mice indicating diastolic dysfunction in the myocardium (Figure 3). Taken together, the pathological cardiac remodeling (functional and structural) in ISO-treated mice are coupled with increased ROS accumulation and ubiquitination of proteins, as shown in immunoblots along with downregulation of mRNA and protein levels of major antioxidants (Figure 4), suggesting the role of isoproterenol-induced oxidative stress in pathological cardiac remodeling.

Previously, others and we have shown that exercise mediated Nrf2 activation increases the antioxidant expression in the myocardium (21–25). However, when the exercise trained heart experienced oxidative stress, the role of Nrf2 and antioxidant signaling in the context of cardiac structure and functional remodeling (systolic vs. diastolic) were not investigated. This study demonstrates decreased Nrf2-ARE binding activity associated with impaired Nrf2/antioxidant signaling in response to isoproterenol administration. Of note, exercise training preserves the transcriptional role of Nrf2 and the trained mice developed resistance to isoproterenol treatment.

Nrf2 increases tolerance against oxidative stress and increases the life span of *Drosophila* by preserving redox homeostasis (58) and also enhances the proliferation of intestinal stem cells (59). Several other studies have also documented that increased oxidative stress leads to decrease in Nrf2 antioxidant cytoprotective mechanisms and increases the progression of the diseases (60–63). In this study, in conjunction with declined Nrf2-signaling, myocardial GSH levels were depleted in response to isoproterenol administration. Furthermore, subsequent to consequences of GSH depletion, increased levels of myocardial ROS were noted. As expected, exercise training stabilized Nrf2/antioxidant signaling, preserving myocardial glutathione levels and prevented ROS accumulation from isoproterenol stress (Figures 5A,C). Thus, prophylactic stabilization of Nrf2 activity and glutathione redox conditions by exercise training facilitates protection against oxidative stress.

Oxidative stress is known to cause various biochemical and conformational modifications in proteins (64–67). Such post-translational modifications (PTMs) of proteins, including the ubiquitination of proteins (68) might result in myocardial toxicity and pathological remodeling. We and others have previously reported that age-associated oxidative stress is coupled with decreased Nrf2/antioxidant signaling and increased oxidative stress and ubiquitination of proteins in the skeletal muscle, atria, and other organs (69–72). However, the role of isoproterenol in protein modifications and subsequent ubiquitination in response to exercise is not well-understood. Here, we show that while isoproterenol increases ubiquitination of proteins, exercise training curtails these anomalies, thereby protecting the myocardium from isoproterenol-induced pathological structural and functional remodeling (Figure 5D). Therefore, a non-pharmacological activation of Nrf2 signaling is likely to protect the heart from oxidative stress induced by isoproterenol.

In conclusion, experiments at 24 h of the last dose of the isoproterenol, the hemodynamics of UNT+ISO demonstrated that increased cardiac output is not inferior to that of MET+ISO mice. Future experiments are planned to test the hypothesis that longer follow-up with UNT+ISO will show significant hemodynamic deterioration compared to MET+ISO. Furthermore, the present study demonstrated that exercise mediated stabilization of functional Nrf2 augments the expression of antioxidant genes and glutathione levels, and protects the myocardium from isoproterenol-induced injury in mice. A major outcome of this study is that chronic, but moderate exercise mediated stabilization of Nrf2 activity enhances endogenous cytoprotective mechanisms, including glutathione redox levels in the myocardium and prevents oxidative stress mediated myocardial injury. Thus, promoting physical activity in human subjects has potential to uphold Nrf2/antioxidant signaling and enhance endogenous cytoprotective mechanisms; this hypothesis warrants further investigation.

## Ethics Statement

This study was carried out in accordance with the recommendations of the Guide for the Care and Use of Laboratory Animals of the National Institutes of Health. The protocol was approved by the Institutional Animal Care and Use Committee (IACUC) at the University of Alabama at Birmingham.

## Author Contributions

The study was conceived/designed by GS and NR. Experiments were performed by GS, AC, SL, BA, and NR. NR, GS, SL and AC interpreted the data and wrote the manuscript. CD, PK, and AD were involved in interpreting the data and critical discussions. All authors read and approved the final version of this manuscript.

### Conflict of Interest Statement

The authors declare that the research was conducted in the absence of any commercial or financial relationships that could be construed as a potential conflict of interest.

## Supplementary Material

Supplemental Figure 1 Exercise stabilizes the systolic heart function. **(A)** Representative m-Mode images from UNT and MET mice. **(B)** Cardiac functional parameters were analyzed using Vevo 3.1 software and represented as a bar graph. *n* = 5–6/group, values are represented as mean ± SD. Significance: ^*^*p* < 0.05;^**^*p* < 0.01; ns, no significance.

Supplemental Figure 2 Exercise stabilizes the diastolic heart function. **(A)** Representative Doppler images captured in pulse wave doppler mode from UNT and MET mice. **(B)** Mitral valve filling velocities (MV E and A) were analyzed using mitral valve images and represented as a bar graph. *n* = 5–6/group, values are represented as mean ± SD. ns, no significance.
