# Supplementary figures and images for: Exercise Mediated Nrf2 Signaling Protects the Myocardium From Isoproterenol-Induced Pathological Remodeling
Source: Front Cardiovasc Med. 2019 Jun 6;6:68. doi: 10.3389/fcvm.2019.00068 (PMC6563599; doi:10.3389/fcvm.2019.00068)

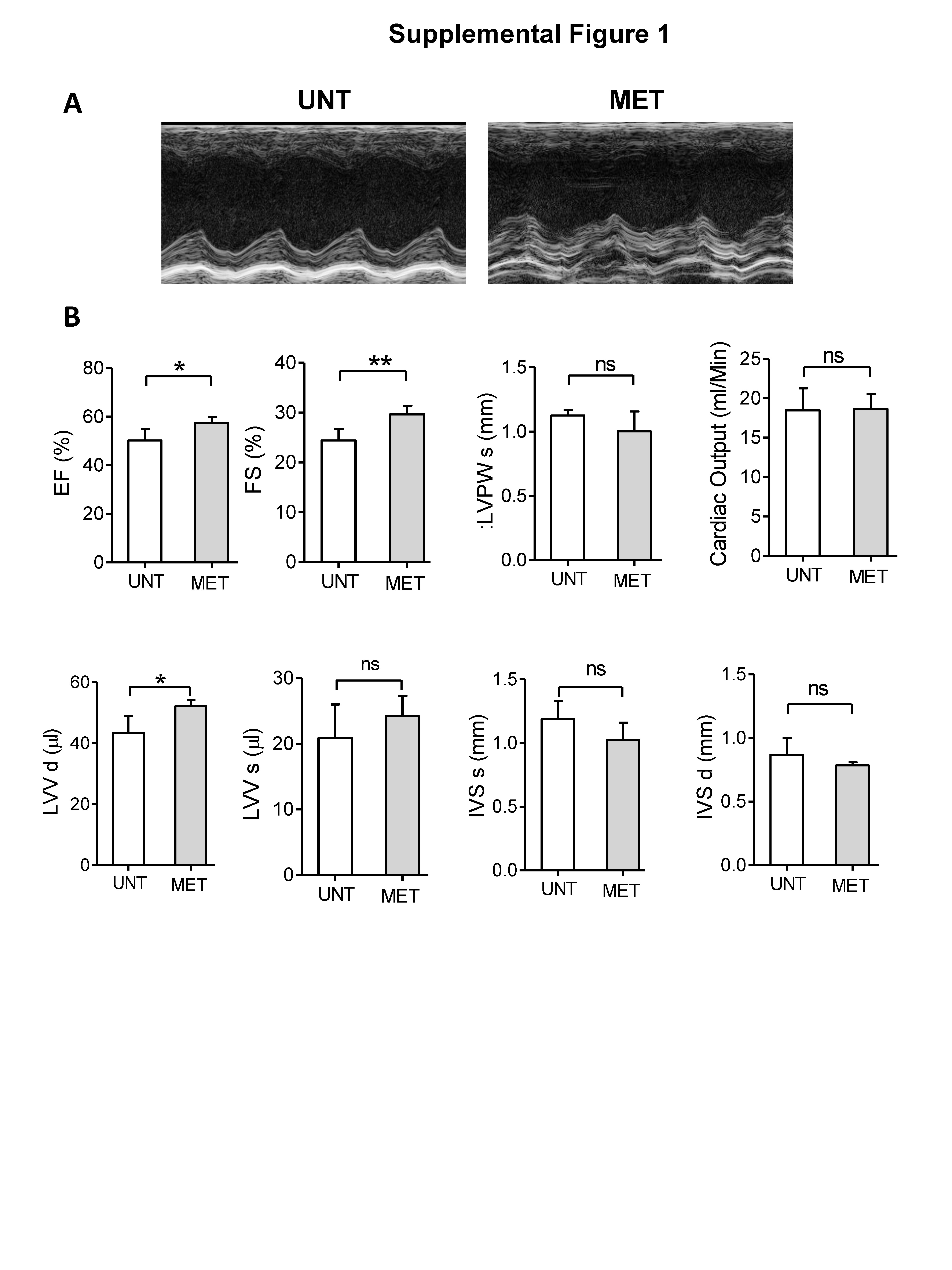

Supplement: Supplemental Figure 1 — Exercise stabilizes the systolic heart function. (A) Representative m-Mode images from UNT and MET mice. (B) Cardiac functional parameters were analyzed using Vevo 3.1 software and represented as a bar graph. n = 5–6/group, values are represented as mean ± SD. Significance: *p < 0.05;**p < 0.01; ns, no significance. [file Image_1.JPEG]

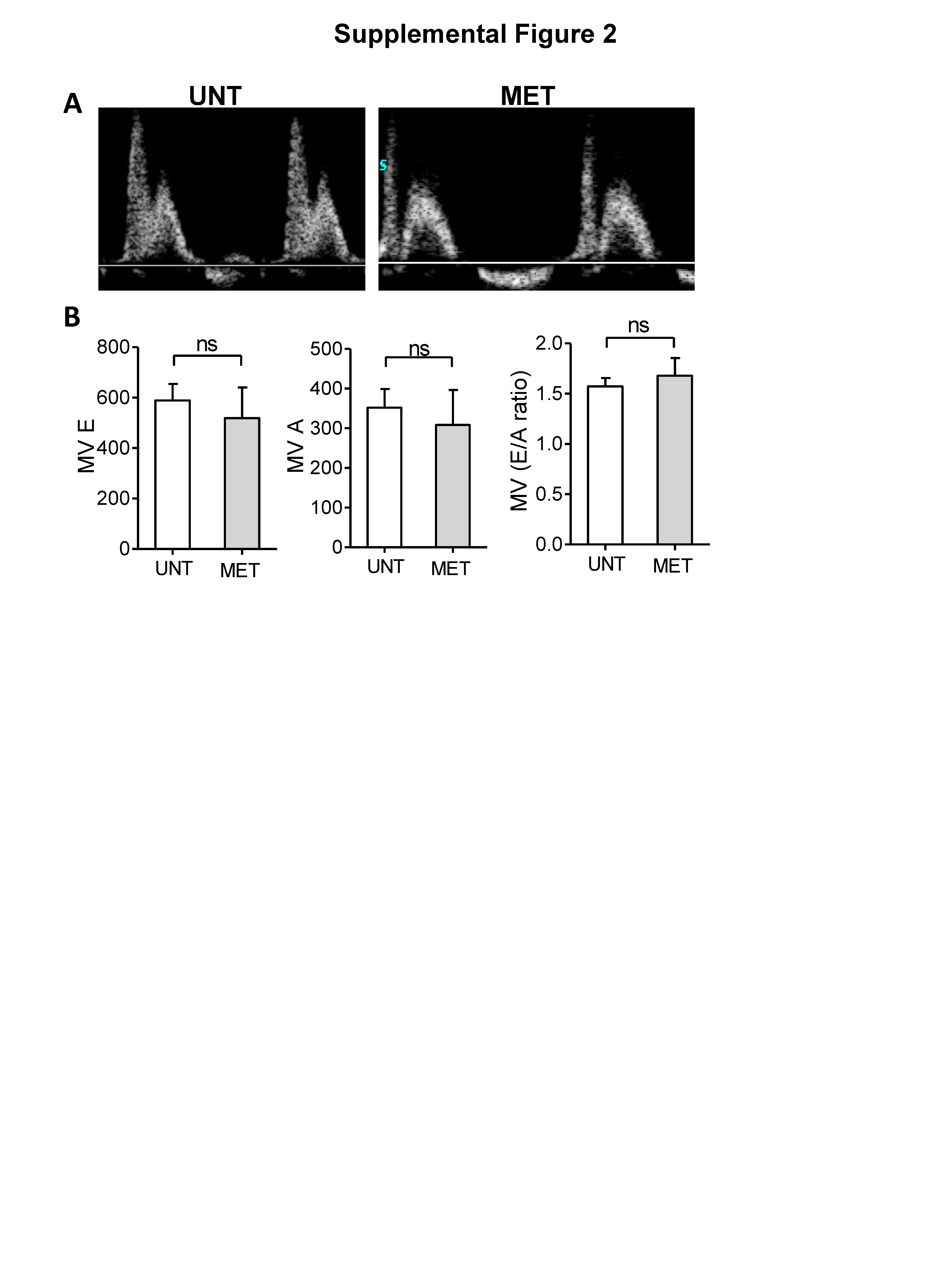

Supplement: Supplemental Figure 2 — Exercise stabilizes the diastolic heart function. (A) Representative Doppler images captured in pulse wave doppler mode from UNT and MET mice. (B) Mitral valve filling velocities (MV E and A) were analyzed using mitral valve images and represented as a bar graph. n = 5–6/group, values are represented as mean ± SD. ns, no significance. [file Image_2.JPEG]
